# Optimization of Detergent-Mediated Reconstitution of Influenza A M2 Protein into Proteoliposomes

**DOI:** 10.3390/membranes8040103

**Published:** 2018-11-08

**Authors:** Catherine H. Crouch, Margaret H. Bost, Tae H. Kim, Bryan M. Green, D. Stuart Arbuckle, Carl H. Grossman, Kathleen P. Howard

**Affiliations:** 1 Department of Physics & Astronomy, Swarthmore College, Swarthmore, PA 19081, USA; meghbost@gmail.com (M.H.B.); tae.kim@utsouthwestern.edu (T.H.K.); 2 Department of Chemistry & Biochemistry, Swarthmore College, Swarthmore, PA 19081, USA; to.green.b@gmail.com (B.M.G.); stuartarbuckle@gmail.com (D.S.A.); carl@brava.com (C.H.G.); khoward1@swarthmore.edu (K.P.H.)

**Keywords:** detergent-mediated reconstitution, integral membrane protein, influenza M2 protein, proteoliposomes, octylglucoside detergent, dynamic light scattering, colorimetric assay

## Abstract

We report the optimization of detergent-mediated reconstitution of an integral membrane-bound protein, full-length influenza M2 protein, by direct insertion into detergent-saturated liposomes. Detergent-mediated reconstitution is an important method for preparing proteoliposomes for studying membrane proteins, and must be optimized for each combination of protein and membrane constituents used. The purpose of the reconstitution was to prepare samples for site-directed spin-labeling electron paramagnetic resonance (SDSL-EPR) studies. Our goals in optimizing the protocol were to minimize the amount of detergent used, reduce overall proteoliposome preparation time, and confirm the removal of all detergent. The liposomes were comprised of (1-palmitoyl-2-oleyl-*sn*-glycero-phosphocholine (POPC) and 1-palmitoyl-2-oleyl-*sn*-glycero-3-[phospho-*rac*-(1-glycerol)] (POPG), and the detergent octylglucoside (OG) was used for reconstitution. Rigorous physical characterization was applied to optimize each step of the reconstitution process. We used dynamic light scattering (DLS) to determine the amount of OG needed to saturate the preformed liposomes. During detergent removal by absorption with Bio-Beads, we quantified the detergent concentration by means of a colorimetric assay, thereby determining the number of Bio-Bead additions needed to remove all detergent from the final proteoliposomes. We found that the overnight Bio-Bead incubation used in previously published protocols can be omitted, reducing the time needed for reconstitution. We also monitored the size distribution of the proteoliposomes with DLS, confirming that the size distribution remains essentially constant throughout the reconstitution process.

## 1. Introduction

Membrane proteins play a critical and often multifaceted role in many biophysical processes. Approximately one third of human genes code for membrane proteins [1]; because membrane proteins are implicated in many diseases, they are the subject of much current research [2]. Many studies of membrane proteins involve reconstituting the proteins into lipid bilayer vesicles, in order to preserve the structure and function of these proteins for studies of structure and/or function in a native-like state [3]. Reproducible methods for the reconstitution of purified membrane proteins into model membranes have been a long-standing challenge [4,5,6,7,8,9,10,11]. The success of a reconstitution protocol is sensitive to the protein, the type of lipids used, and the choice of detergent [6]. Thus, the optimization of the reconstitution strategy for each new experimental system is necessary for the collection of reproducible high-quality data. 

In this article, we report the optimization and detailed, rigorous physical characterization of detergent-mediated reconstitution by direct insertion into pre-formed liposomes of an integral membrane protein that is of considerable current interest, namely influenza M2 protein [12,13]. The optimization is guided by literature on the mechanism of the direct insertion process [4,5] and is designed to completely remove all detergent and minimize long incubation times that can lead to sample degradation such as spin-label detachment. Our liposomes are made up of lipids used in a range of published M2 papers [14,15,16], 1-palmitoyl-2-oleyl-*sn*-glycero-phosphocholine (POPC) and 1-palmitoyl-2-oleyl-*sn*-glycero-3-[phospho-*rac*-(1-glycerol)] (POPG), POPC/POPG 4:1 molar ratio.

The M2 protein is a membrane-bound protein that is essential both to the uncoating of virions when viruses enter cells [17] and the creation of curvature critical to viral budding. An atomic-level understanding of the role M2 plays in viral assembly and budding process could lead to strategies to inhibit the replication of viruses and new tactics for inhibiting viral infectivity. The threat of future pandemics, coupled with the growing resistance to existing antivirals, makes the development of new antiviral influenza drugs a national healthcare priority. A range of biophysical techniques, including x-ray crystallography [18], solution NMR [19], solid state NMR [20,21] and site-directed spin labeling electron paramagnetic resonance (SDSL-EPR) [22,23,24,25] have been used to determine atomic level details of the conformation of the M2 protein. Due to the sample requirements of the different structural methods and the hypotheses being tested, the conformation of M2 has been probed using constructs ranging from 25 amino acid truncations up to the 97-amino acid full-length protein. Different hydrophobic membrane mimics have been employed, ranging from detergent micelles [19] to liposomes comprised of viral mimetic lipid mixtures [26]. Depending on the structural method, the construct of the M2 protein, and the choice of membrane mimic, different strategies for protein reconstitution have been employed. 

Two classes of reconstitution strategies have been used to prepare M2 proteoliposomes for structural studies: organic co-solubilization of lipids/M2 [24] and detergent-mediated reconstitution of M2 by incubation with pre-formed vesicles partially or completely solubilized with detergent [27]. To avoid the complication of residual organic solvent remaining in the proteolipsomes and concerns about refolding of organic solvent-solubilized protein, we chose to use detergent-mediated reconstitution to incorporate the spin-labeled M2 protein into liposomes. Our reconstitution uses octylglucoside (OG) detergent, a nonionic detergent widely used for membrane proteins. It is suitable for our work because OG has been established to facilitate direct insertion of protein into preformed, detergent-saturated liposomes, which has the advantage of promoting unidirectional insertion [5], and it is easy to remove because of its high critical micelle concentration (CMC) and small micelle size [6,28,29]. However, care must be taken to fully remove nonionic detergents, as otherwise they can deactivate the protein. 

In previously published M2 studies that employ detergent-mediated reconstitution for preparation of proteoliposomes, two different strategies have been used to remove residual detergent: (1) Dialysis of the protein-detergent mixture against detergent free-buffer over a period of days [30,31] and (2) adsorption of detergent by macroporous polymeric beads (Bio-Beads) [15,27] over a period of hours. We removed the detergent in our work with Bio-Beads because it takes significantly less time than dialysis. Prolonged sample preparation increases the likelihood of sample degradation over time; for example, in studies that require the attachment of a reporter group, such as in SDSL-EPR studies, the potential instability of probe-protein bonds can lead to a decrease over time in the overall labeling of the protein and a concurrent increase in free reporter, which can significantly compromise data analysis [32]. Furthermore, proteoliposomes can precipitate over time. Lengthy dialysis times therefore have the potential to significantly degrade the quality of samples. 

## 2. Materials and Methods 

### 2.1. Expression, Purification and Spin-Labeling of M2 Protein 

The expression and purification of the full-length M2 protein followed published optimized protocols [15]. For use in site-directed spin labeling electron paramagnetic resonance spectroscopy (SDSL-EPR), a spin label was covalently linked via a disulfide linkage to a cysteine residue placed at site 51 as previously described [15]. The spin label used was (1-Oxyl-2,2,5,5-tetramethyl-∆3-pyrroline-3-methyl) methanethiosulfonate, MTSL (chemical structure provided in Figure 4a; made by Toronto Research Chemicals, North York, ON, Canada). 

### 2.2. Liposome Preparation

Liposomes were prepared with a 4:1 molar ratio of 1-palmitoyl-2-oleyl-*sn*-glycero-phosphocholine (POPC, Avanti Polar Lipids, made by Avanti, Alabaster, AL, USA) and 1-palmitoyl-2-oleyl-*sn*-glycero-3-[phospho-*rac*-(1-glycerol)] (POPG, Avanti Polar Lipids). Chloroform solutions of POPC and POPG, each 25 mg/mL, were combined in a 4:1 molar ratio. For each sample, 130 µL of the mixture was placed in a glass vial; the chloroform was removed under a gentle stream of nitrogen and then the lipid films were placed under high vacuum overnight. Each lipid film was hydrated in 350 µL of buffer (50 mM Tris pH 8, 100 mM KCl, 1 mM EDTA) for 30 min and then vortexed for 2 min to resuspend the film; 250 µL of the resulting suspension was then extruded 15 times through a 200 nm polycarbonate filter using an Avanti Mini-Extruder that had been previously hydrated with the same buffer. The resulting liposome sample has an average effective diameter (determined by DLS) of 180 nm with a polydispersity (the second moment of the distribution determined by DLS, see Section 2.5) of 17% and a concentration of 6.0 mg/mL (accounting for buffer filling the body of the extruder from the hydration process). The liposome sample was then diluted to 2.0 mg/mL for the saturation point determination and the reconstitution.

To maintain a reproducible liposome concentration, which is important in reliably adding detergent to the saturation point, the extruder must be disassembled and rinsed with buffer and a new filter used for each sample. This is required because the volume within the body of the extruder (as opposed to the syringes) affects the final concentration, and it makes a difference whether it is filled with buffer or with liposome solution remaining from previous extrusions.

### 2.3. Reconstitution of M2 Protein into Liposomes 

**Figure 1:**
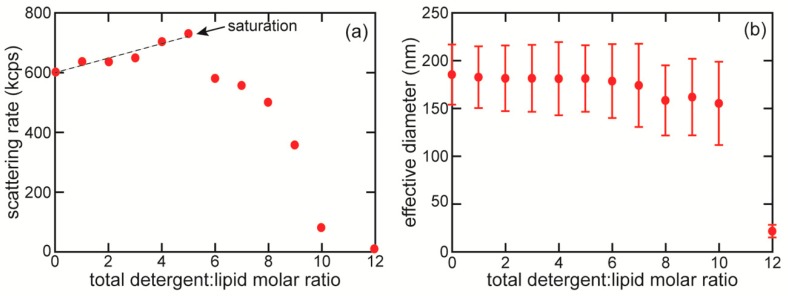
Determination of amount of detergent needed to saturate liposomes. (**a**) DLS count rate (photon scattering rate) vs. total molar detergent:lipid ratio, for OG detergent and 4:1 POPC:POPG liposomes (lipid concentration 2.0 mg/mL, corresponding to 2.6 mM). The dashed line shows a linear best fit to data points up to saturation. The saturation point corresponds to the significant drop in scattering. Uncertainty in count rate (determined by variation between 2 measurements per OG concentration) is 4 kcps, much smaller than the data markers. (**b**) Average hydrodynamic diameter of particles (liposomes); error bars calculated from the polydispersity of the distribution.

Here we summarize our optimized reconstitution protocol; in Section 3, we present the detailed work leading to this protocol. As described below in Section 3.2 and shown in Figure 1, we determined the detergent saturation point for our liposome preparation to correspond to a total molar detergent to lipid ratio of roughly 5:1. The liposomes were brought to slightly past the saturation point by adding 50 mM Tris pH 8, 100 mM NaCl, 6.13 mg/mL OG (Carbosynth Limited, San Diego, CA, USA). In our preparation, for each liposome sample, 200 µL of liposomes was combined with 400 µL of OG solution to produce a lipid concentration of 2.0 mg/mL. The lipid/detergent solution was allowed to equilibrate for 30 min. 

Next, a protein-detergent mixture was prepared and added to the liposomes to achieve a protein to lipid molar concentration ratio of 1:500. The detergent concentration in this mixture is set to provide one micelle for each protein tetramer; the required concentration must be calculated for each preparation, taking into account the protein and detergent concentrations in the original protein preparation, the critical micelle concentration for OG (19–25 mM), and the mean aggregation number for OG micelles (approximately 90) [28]. 

A slurry of hydrophobic polystyrene beads (Bio-Beads SM-2, Bio-Rad Laboratories, Hercules, CA, USA) was prepared by adding buffer (50 mM Tris pH 8, 100 mM NaCl) dropwise to the beads until they were hydrated. The Bio-Bead slurry was stirred slowly and degassed under vacuum for 1 h. While the proteoliposome sample was gently nutated at 4 °C, a series of six 50 µL aliquots of the Bio-Bead slurry were added at 15 min intervals. 

**Figure 2:**
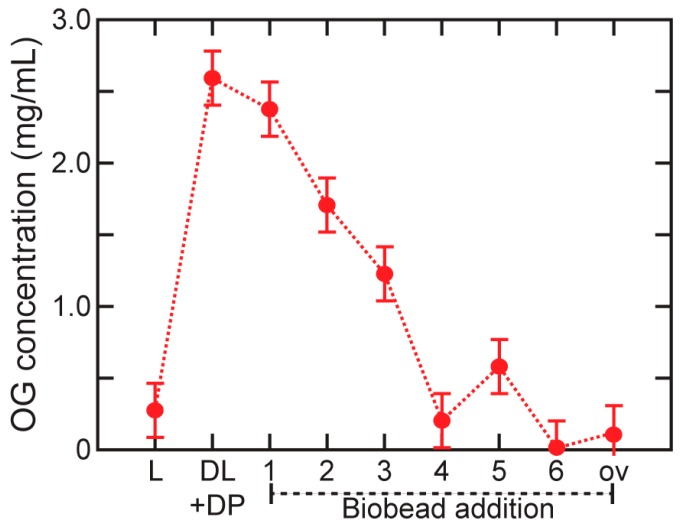
Detergent concentration at each step of reconstitution. OG concentration determined by a colorimetric assay at each step of the reconstitution of full length influenza M2 into POPC:POPG 4:1 liposomes. Time points at which measurements were made: L = liposomes before any addition of detergent; DL + DP = detergent-saturated liposomes mixed with protein-detergent micelles, before detergent removal; 1–6 = after incubation with the corresponding Bio-Bead aliquot; ov = after overnight incubation with a 7th Bio-Bead aliquot.

**Figure 3:**
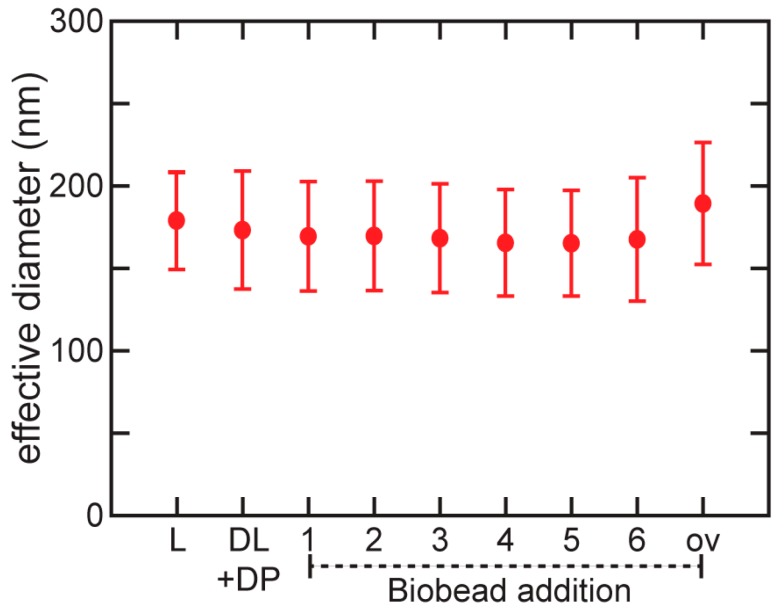
Liposome size distribution throughout reconstitution. Effective liposome diameter at each step of OG detergent addition and removal process for reconstitution of full length influenza M2 into POPC:POPG 4:1 liposomes. Error bars showing the width of the distribution are calculated from the polydispersity determined from cumulant analysis (see Methods). Time points at which measurements were made are the same as in Figure 2.

In a previously published protocol for reconstitution of M2 [15], a final 200 µL aliquot of Bio-Beads is added to the proteoliposome solution and nutated overnight at 4 °C. We included the overnight step in the experiments used to monitor detergent removal (data presented in Figure 2 and Figure 3), and found that it is not necessary for removing all detergent, so this step is omitted from the final optimized protocol. The proteoliposome solution was decanted from the Bio-Beads and concentrated to a spin-labeled protein concentration of approximately 100 µM using Amicon Ultra-0.5 Centrifugal Filter Devices at 13,000× *g*. With a protein to lipid ratio of 1:500 in the sample, the average number of protein tetramers per proteoliposome is on the order of 150.

### 2.4. Colorimetric Assay to Quantify Detergent Concentration

The determination of the concentration of OG followed a published colorimetric procedure [33,34], with minor modifications as described below. 230 µL of the OG-containing sample was mixed with 25 µL of 20% 2,6 dimethylphenol (Aldrich D174904-5G, Sigma-Aldrich, St. Louis, MO, USA) in absolute ethanol (Pharmco-Aaper 111000200, 0.789 g/mL, Greenfield Global, Brookfield, CT, USA) and 750 µL of concentrated sulfuric acid (Baker Analyzed ACS reagent H48F01, Avantor, Radnor, PA, USA), allowing to incubate at room temperature for 40 min, and then measuring the absorbance of the resulting solution at 510 nm in a quartz cuvette. The absorbance was then related to concentration by comparing to a calibration curve determined with a series of concentrations of OG in a background solution containing the same materials (lipid, protein and buffer) as the samples to be tested. We found that it was important to complete an OG calibration curve in the presence of the lipid/protein/buffer background as the background signal can distort an accurate measurement of OG concentration [33]. 

### 2.5. Dynamic Light Scattering

Light scattering autocorrelation functions were recorded at room temperature using a Brookhaven Instruments BI-200SM goniometer (Brookhaven Instruments, Holtsville, NY, USA), a BI-9000AT correlator (Brookhaven Instruments), and 9KDLSW software. The sample was illuminated by an Ar-Kr laser operating at 514.5 nm. The scattering angle was 90°, and both the incident and scattered light passed through linear polarizers perpendicular to the scattering plane. The laser power was set at 15 mW; the aperture in front of the detector was kept at its minimum size to maintain a count rate well below 1 Mcps (cps = counts per second). The autocorrelation functions recorded by the correlator software were analyzed using the method of cumulants, with the effective diameter (based on the Stokes-Einstein relation) and polydispersity (fractional width of the distribution) calculated from the first and second cumulants, respectively. 

A 100 µL aliquot of each sample was thoroughly mixed by pipetting and then placed in a glass capillary tube for measurement. Lipid concentration was approximately 2.0 mg/mL in the same buffer used for liposome preparation. All buffers were filtered to exclude dust, and the “dust filter” setting on the correlator was used to reject further bursts of intensity from large particles. The temperature was monitored and provided as an input to the analysis software; samples were allowed to equilibrate to the temperature of the apparatus for at least 30 min. Each measurement lasted for 2 min with a count rate of between 500 and 900 kilocounts/s (depending on the specific sample); each sample was measured twice (more if the two measurements were not within typical variation of 2–4 kcps) and the results averaged. 

### 2.6. EPR Spectroscopy

Continuous wave (CW) EPR spectra were recorded at room temperature on an X-band Bruker EMX spectrometer equipped with an ER4123D resonator. Samples used for analysis of spectral line shapes were placed in glass capillary tubes and EPR spectra were acquired using 2 mW incident microwave power, 1 G field modulation amplitude at 100 kHz, and 150 G sweep width. For comparison of line shapes, each spectrum was double integrated and normalized to the same number of spins. 

## 3. Results: Optimization of OG-Mediated Reconstitution of Influenza M2 into Liposomes

### 3.1. Overview of Direct Insertion into Detergent-Saturated Liposomes Using OG

A range of approaches to detergent-mediated reconstitution (DMR) of membrane proteins have been used [4,5,6,7,8,9,10,11,28,35,36,37]. In the work reported here, we optimized a protocol for direct insertion of M2 into detergent-saturated liposomes; this approach has been demonstrated with several different membrane proteins to give efficient and largely unidirectional reconstitution [4,5], while using minimal amounts of detergent. The alternative approach of reforming proteoliposomes from mixed micelles [7,8,38] can produce high protein concentrations, but as we seek a final protein:lipid ratio of 1:500, this is not necessary. In addition, we directly quantify detergent concentration throughout the removal process to ensure that all detergent is removed prior to subsequent experiments with the proteoliposomes. We use octylglucoside (OG), as that detergent has been widely used when reconstituting full length influenza M2 protein [15,30,35] and facilitates direct insertion [4,5].

The direct insertion process involves the following steps:

1. preparing liposomes of a defined size, in our case by extrusion;
2. adding detergent to the liposomes until they are saturated with detergent monomers;
3. adding protein-detergent micelles to the detergent-saturated liposomes, and
4. removing the detergent.

The optimization process requires determining the amount of detergent needed to saturate the liposomes, and determining when the detergent is entirely removed. In the following sections we explain the importance of these steps.

### 3.2. Detergent-Liposome Saturation Point Determination

The effect of detergent on the structure of the liposomes depends on the detergent concentration. At very low concentrations of detergent, individual detergent monomers insert into the liposomes; as the detergent concentration increases, more monomers are inserted, until the liposomes are saturated with detergent molecules. This condition is known as the saturation point. If the detergent concentration increases beyond the saturation point, the saturated vesicles are gradually broken down into small lipid-detergent micelles. The condition at which all liposomes have been converted to mixed micelles is called the solubilization point [5,6,11].

With certain choices of detergent, including OG, proteins can be directly incorporated into detergent-saturated liposomes, rather than requiring complete solubilization of the liposomes [4,5,6]. This direct insertion approach, in which rapid equilibration between protein-detergent micelles and detergent-saturated liposomes result in protein incorporation into the liposomes, has three significant advantages. First, direct insertion incorporates the protein in the proper orientation at a high rate (85–90%) [5], while solubilizing and then reforming the liposomes leads to randomly oriented proteins. Second, direct insertion maintains the liposome size distribution throughout the process, as our own results confirm, while reforming liposomes after complete solubilization produces a more heterogenous size distribution, which may have a different average size than the original liposomes [4,5]; reforming after solubilization also increases the number of multilamellar vesicles [39]. Finally, minimizing the amount of detergent used is valuable in its own right, as large excesses of detergent require more absorbent to completely remove the detergent, as well as potentially prolonging the removal process and allowing the proteolipsomes to deteriorate. Furthermore, OG detergent is a costly reagent, so minimizing its use has the added benefit of reducing the cost of experiments.

As the concentration of detergent corresponding to the saturation point and the solubilization point depend on the lipid concentration [10,11,40], it is necessary to determine the saturation point not only for the particular choice of detergent and liposome composition, but also for the particular lipid concentration used. We used the scattering intensity from dynamic light scattering (DLS) to determine the saturation point of the detergent-liposome mixture. DLS is commonly used to determine the size distribution of particles in solution from the autocorrelation function of the time dependent scattered light intensity [41]. An advantage of using DLS rather than turbidimetry to determine the saturation point is that DLS also allows determining the liposome size distribution before and during the reconstitution process. After detergent removal, DLS can be repeated to determine the final size distribution of the liposomes.

Figure 1a shows the time-averaged scattering count rate from mixtures of OG and 4:1 POPC:POPG liposomes (with 2.0 mg/mL lipids), vs. OG concentration, with the saturation point labeled. In our approach, the scattering rate provides the same information as the optical density in traditional turbidimetry. At detergent concentrations below the saturation point, the liposomes absorb detergent, but the total number of scattering particles in solution remains constant, so the count rate increases only slightly with increasing detergent concentration. As the detergent concentration increases past the saturation point, the liposomes are steadily broken down into much smaller micelles, which scatter much less, and therefore the count rate decreases steeply, until the liposomes are completely solubilized and the count rate reaches nearly zero. Turbidimetry measurements have established that the saturation point occurs at a significant drop in scattering [4,5]; our data below saturating detergent concentrations are marked with a best-fit line to serve as a guide to the eye. From these data, we determined the saturation point to occur at or slightly above 5:1 total detergent to lipid molar ratio, corresponding to 13–14.5 mM OG. We repeated this process three times and obtained consistent results each time. In our optimized protocol we used 15.2 mM OG in order to be slightly above the saturation point. While this value for the saturation point is somewhat lower than that found by Paternostre et al. (~19 mM OG) using egg phosphatidylcholine liposomes at a similar lipid concentration [42], there is disagreement in the literature [5,10,11,42] over whether at low detergent and lipid concentrations, aqueous detergent concentrations may depend on the concentration of and the identity of the lipids, which could explain the discrepancy.

DLS also provides the average hydrodynamic diameter of the scattering particles, which we can use to confirm our interpretation of the scattering count rates. We found that the average hydrodynamic diameter is essentially constant up to the saturation point, and then decreases slightly, accompanied by an increase in polydispersity, until the liposomes are completely solubilized, when the average diameter plummets to nearly zero (Figure 1b). Because the scattering intensity from particles in solution measured by DLS is proportional to the sixth power of the particle radius [41], the lipid-detergent micelles contribute very little to the signal. Consequently, the average size decreases only modestly as the amount of detergent increases somewhat past the saturation point, and does not decrease substantially until a significant fraction of the liposomes have been converted to mixed micelles [10]. 

### 3.3. Characterization of Detergent Removal

Complete removal of detergent from proteoliposomes is critically important to a wide variety of experiments, because residual detergent can compromise or inhibit protein function by denaturing the protein or stabilizing non-functional conformations [6]. Two widely used methods for removing detergent are dialysis or by the use of macroporous polymeric adsorbents composed of a large number of highly crosslinked polystyrene microspheres called Bio-Beads (Bio-Beads SM-2, Biorad) [6,29,38]. Bio-Beads present a high surface area for adsorption of nonpolar substances or surface-active agents from aqueous solutions [6,43]. The amount of Bio-Beads needed varies by detergent, lipids, and conditions [43], producing a need to optimize the use of Bio-Beads for detergent removal for each set of sample conditions. 

For this study, we initially tested a previously published protocol for the reconstitution of M2 into liposomes [15], in which six 50 µL aliquots of Bio-Beads are successively added and incubated for 15 min each; a seventh 200 µL aliquot is then added and incubated overnight. (Another protocol used six Bio-Bead aliquots added at 16 min intervals with a final incubation of 90 min after the final addition [27].) After each incubation with a new aliquot of Bio-Beads, we withdrew an aliquot of the sample, which we used both for DLS characterization of the liposome size distribution and colorimetric measurement of OG concentration.

Figure 2 shows OG concentration measured at each step of detergent addition and removal, using a 2,6 dimethylphenol colorimetric assay [34]. These measurements indicate that the overnight incubation is unnecessary; the OG may be fully removed after as few as four 15-min incubations, and is completely removed after six.

Figure 3 shows the effective diameter of the liposome distribution for the reconstitution with protein, measured using light scattering. We found that the average effective diameter of the liposome distribution remains essentially constant throughout the process, confirming that direct insertion of the protein in detergent-saturated liposomes maintains the liposome size distribution. The breadth of the distribution, represented by the error bars, increases slightly with the initial addition of detergent and thereafter remains essentially constant, consistent with previous work [44].

### 3.4. EPR Data from M2 Reconstituted into Liposomes Using Optimized Protocol

**Figure 4:**
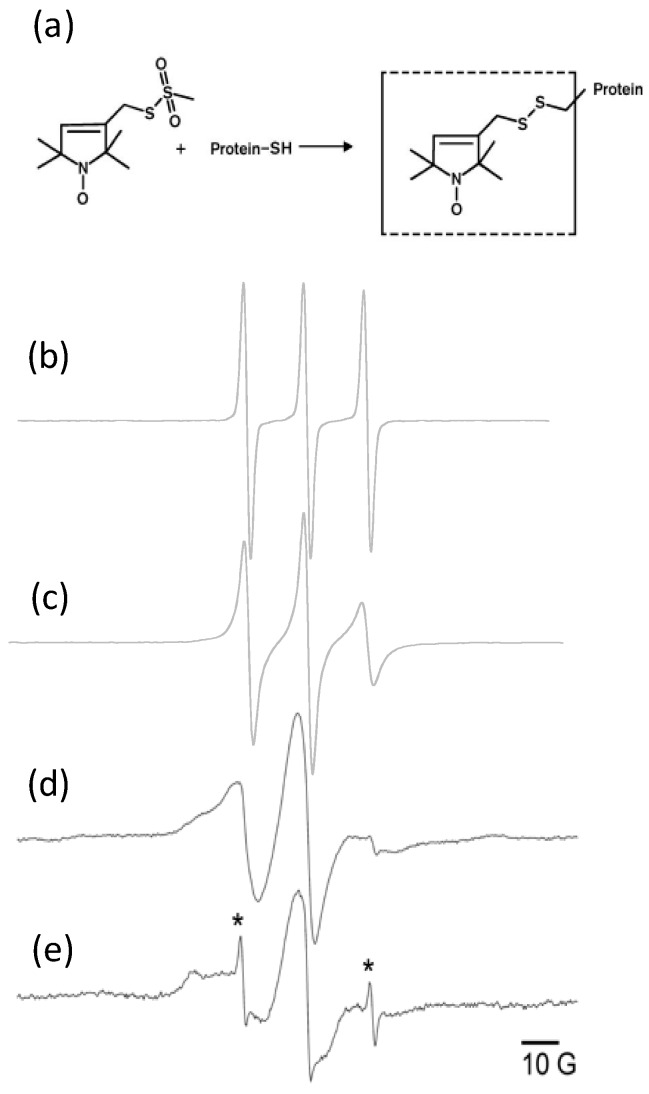
Spin-label structure and EPR spectra of proteoliposomes. (**a**) The nitroxide spin-label, 1-Oxyl-2,2,5,5-tetramethyl-∆3-pyrroline-3-methyl) methanethiosulfonate (MTSL), is covalently linked to a cysteine (site 51) via disulfide linkage. CW X-band EPR spectra of (**b**) MTSL spin label in aqueous solution (**c**) MTSL spin label attached to full-length M2 protein solubilized in OG detergent micelles (**d**) MTSL spin label attached to full-length M2 protein that has been freshly reconstituted into 4:1 POPC:POPG liposomes and (**e**) same sample as d collected one week after preparation showing the presence of free spin label. * indicate spectral components arising from free spin label in sample (**e**). Samples (**c**–**e**) prepared in 50 mM Tris pH 8, 100 mM NaCl.

To examine whether minimizing preparation time leads to improved sample properties, as well as to confirm complete insertion of the protein into the liposomes, we carried out SDSL-EPR on reconstituted liposomes either immediately after preparation or after an additional incubation. SDSL-EPR measurements are most accurate when performed on proteoliposomes with all detergent removed and with negligible amounts of free spin label. Free spin-label produces a relatively sharp three-line spectrum, whereas a spin label attached to a membrane protein reconstituted into bilayer liposomes produces a broader spectrum due to its restricted motion [45]. Consequently these measurements are a sensitive probe of the presence of any protein not inserted into liposomes. Figure 4a shows the chemical structure of the spin label and its attachment to the protein.

Figure 4b shows the spectrum from the MTSL spin label alone (not attached to protein) in aqueous solution. Figure 4c shows the spectrum from spin labeled M2 protein solubilized in OG micelles, with no lipids present; this spectrum is slightly broadened compared to the label alone, indicating minimal restriction of the spin label mobility. Figure 4d presents the spectrum of spin labeled M2 in proteoliposomes freshly prepared with our optimized protocol. The breadth and shape of the spectrum in Figure 4d is typical of a spin label bound to a membrane-bound protein [15]. Furthermore, Figure 4d demonstrates that our proteoliposome spectrum does not show evidence of the spectral signature of M2 in detergent micelles shown in Figure 4c. This is consistent with Figure 2, which demonstrates via a colorimetric assay that OG detergent is no longer detectable at the end of our optimized reconstitution method. The battery of EPR experiments we carry out on our proteoliposome samples [15,22], including accessibility to paramagnetic relaxation agents and double electron-electron resonance, can take several days, so the ability to eliminate the final 12-h incubation is very advantageous. As previously stated, we chose to remove the detergent with Bio-Beads because dialysis times for the removal of detergent in M2 reconstitution have been reported to last for several days [31,46].

To highlight the importance of minimizing proteoliposome preparation time for samples with labile spectroscopic tags, we show spectrum 4e, which demonstrates what typically happens to proteoliposomes over several days. This spectrum was obtained from a sample of the same proteoliposomes measured in Figure 4a, after a week of storage at 4 °C. The superimposed sharp line spectrum is consistent with the presence of spin label that is no longer bound to the protein. The breaking of label-protein bonds decreases the extent of labeling of the protein, reducing sensitivity and complicating spectral analysis [32]. Our optimized reconstitution protocol enables the complete removal of detergent in 1.5 h of incubation with Bio-Beads. 

## 4. Discussion and Conclusions

We have optimized the procedure for reconstitution of influenza M2 protein by direct insertion into OG detergent-saturated 4:1 POPC:POPG liposomes. Specifically, we determined the amount of OG detergent needed to saturate the liposomes, allowing the minimum amount of detergent to be used, and demonstrated by means of a colorimetric analysis that a 90-min incubation with Bio-Beads (6 aliquots of Bio-Beads for 15 min each) is sufficient to completely remove the OG detergent. Previously published protocols include an additional 75 min of incubation [27] or a final overnight incubation [15]; our colorimetric analysis indicates these are not necessary. Reducing the time needed to remove detergent significantly improves experimental efficiency and allows collection of SDSL-EPR data before possible spin label detachment and/or aggregation of proteoliposomes. Furthermore, we have confirmed using light scattering that the size distribution of the proteoliposomes after detergent removal is very similar to that of the original liposomes.

Optimization of the membrane reconstitution protocol for influenza A M2 protein is critical for reproducible biophysical studies that report on the conformation and dynamics of this protein. Influenza A M2 protein orchestrates several essential events in the viral life cycle, including viral assembly and budding [12,13]. An atomic-level understanding of the viral assembly and budding process could lead to strategies to inhibit the replication of viruses and new tactics for inhibiting viral infectivity. The threat of future pandemics, coupled with the growing resistance to current antivirals, makes the development of new antiviral influenza drugs a national healthcare priority.
